# Public Valuation of Direct Restorations: A Discrete Choice Experiment

**DOI:** 10.1177/00220345221108699

**Published:** 2022-07-25

**Authors:** O. Bailey, S. Stone, L. Ternent, C.R. Vernazza

**Affiliations:** 1 School of Dental Sciences, Newcastle University, Newcastle upon Tyne, UK; 2 Population Health Sciences Institute, Newcastle University, Newcastle upon Tyne, UK

**Keywords:** dental caries, operative dentistry, dental economics, patient preference, healthcare policy, health services research

## Abstract

Direct posterior dental restorations are commonly provided following management of dental caries. Amalgam use has been phased down and the feasibility of a phase-out by 2030 is being explored. Alternative direct restorative materials differ in their outcomes and provision. This research aimed to elicit the UK population’s preferences for different attributes of restorations and their willingness to pay (WTP) for restorative services and outcomes. A discrete choice experiment (DCE) was designed with patient and public involvement and distributed to a representative sample of the UK general population using an online survey. Respondents answered 17 choice tasks between pairs of scenarios that varied in levels of 7 attributes (wait for filling, clinician type, filling color, length of procedure, likely discomfort after filling, average life span of filling, and cost). An opt-out (no treatment) was included. Mixed logit models were used for data analysis. Marginal WTP for attribute levels and relative attribute importance were calculated. In total, 1,002 respondents completed the DCE. Overall, respondents were willing to pay £39.52 to reduce a 6-wk wait for treatment to 2 wk, £13.55 to have treatment by a dentist rather than a therapist, £41.66 to change filling color from silvery/gray to white, £0.27 per minute of reduced treatment time, £116.52 to move from persistent to no postoperative pain, and £5.44 per year of increased restoration longevity. Ability to pay affected willingness to pay, with low-income respondents more likely to opt out of treatment and value restoration color (white) and increased longevity significantly lower than those with higher income. Clinicians should understand potential drivers of restoration choice, so they can be discussed with individual patients to obtain consent. It is important that policy makers consider general population preferences for restorative outcomes and services, with an awareness of how income affects these, when considering the potential phase-out of amalgam restorations.

## Introduction

Direct restorations are frequently placed following the operative management of dental caries. Worldwide, more than 1.1billion direct restorations were placed in 2015 (S. Heintze, personal communication, 2022) and amalgam has long been an effective, low-cost material (Norwegian Climate and Pollution Agency 2012). An amalgam phase-down has been implemented (Regulation (EU) 2017/852 2017) following a global convention (Minamata Convention on Mercury 2013), and the feasibility of a complete phase-out by 2030 is currently being assessed. The World Health Organization has stated that “systematic studies on the economic and social costs and benefits of quality mercury-free materials have not yet been published” (Fisher et al. 2018). Understanding these social costs and benefits in relation to existing materials is critical when planning patient-centered service provision (Listl et al. 2022).

English National Health Service primary care expenditure on amalgam in permanent teeth was crudely estimated at £200 to £300 million in 2015–2016 (C. Vernazza and K. Carr, personal communication, 2018). A 2019 survey of UK clinicians indicated that amalgam remains the most used material in permanent molars, with only 6.7% respondents using no amalgam. Clinicians took longer to place composite compared to amalgam, charged more, reported an increased incidence of postoperative complications, and were much less confident placing composite in difficult situations (Bailey et al. 2022a, 2022b). Systematic reviews indicate the superior longevity of amalgam restorations in permanent posterior teeth compared to composite (Khangura et al. 2018; Worthington et al. 2021).

Economic valuation of restorative dental care commonly focuses on a single outcome, such as the life span of a restoration or tooth (Smales and Hawthorne 1996; Tobi et al. 1999; Khangura et al. 2018; Schwendicke et al. 2018). Patient or public valuation of the importance of these parameters is not commonly sought (Listl et al. 2022), and other important factors are often not considered, including the aesthetic outcome, process of care considerations (e.g., how long the treatment would take), or out-of-pocket monetary costs.

Stated preference techniques are used to elicit preferences where consumer/patient behavior in the real world cannot be relied upon to provide an accurate representation of preferences. This lack of reliability is inevitable where imperfect free-market economies exist, as is commonly the case in health care. Discrete choice experiments (DCEs) are a stated preference technique based on assumptions, underpinned by economic theory (Lancaster 1966) and random utility theory (McFadden 1973), that health care services can be described by their characteristics (attributes) and that individuals value services depending on the levels of these attributes (Ryan 2004). DCEs are well established in valuing health interventions (Clark et al. 2014) but have been sparsely used in dentistry (Barber et al. 2018). Although the inclusion of cost attributes in valuing health care is perhaps controversial (Bryan and Dolan 2004), their use in dentistry is less so as the public is often used to paying for dental treatment (Boyers et al. 2021). After relevant attributes are determined, a hypothetical survey is carried out where respondents make choices between a series of pairs of alternatives with different levels of the relevant attributes. This allows the relative importance of the levels of the attributes to be estimated, alongside marginal willingness to pay (mWTP) values for each attribute level (where a cost attribute is included).

A previous DCE looked at the importance of restoration longevity, color, and adverse outcomes to young patients and dental professionals (Espelid et al. 2006). However, the results had limited scope to inform policy given the framing and sampling of the survey, which was confined to specific groups in Norway and Denmark. In addition, no cost attribute was included, meaning mWTP values could not be calculated.

The aim of this study was therefore to understand the UK general public’s preferences for directly placed restorations in posterior permanent teeth. The objectives were to quantify 1) mWTP values for the differing levels of the attributes, 2) the relative attribute importance (RAI), and 3) any differences in these based on income subgroups.

## Method

The study was carried out and reported in accordance with available guidance (Bridges et al. 2011; Johnson et al. 2013; Hauber et al. 2016; Staniszewska et al. 2017). A favorable ethical opinion was obtained from Newcastle University Research Ethics Committee (2320/2020).

### Attribute and Level Selection

A scoping literature review revealed 1 previous DCE valuing aspects of posterior dental restorations (Espelid et al. 2006). It was of limited use in designing this DCE due to the framing and attribute selection. Patient and public involvement (PPI) guided attribute and level selection through an online focus group (Coast et al. 2012) (participants recruited through VOICE [www.voice-global.org]). A short contingent valuation exercise using an online bidding game was undertaken with the focus group participants to inform cost attribute levels. This was used alongside recent cross-sectional data from UK dentists and therapists (Bailey et al. 2022a), the clinical evidence base (Khangura et al. 2018), and research group discussions, which included expert opinion from dental specialists to determine relevant attributes and levels for inclusion in the survey. This ensured that the attributes and levels were clinically meaningful and relevant to the general public and policy-makers. Initial attribute levels were modified following piloting, resulting in the following attributes and levels:

Waiting time for filling: 0, 2, 4, 6 wk
Clinician type: dentist, dental therapist
Filling color: white, silvery-gray
Length of filling procedure: 20, 40, 60, 80 min
Likely discomfort after filling: none, mild, moderate, persistent
Average life span of filling: 5, 8, 11, 14 y
Cost: £15, £25, £35, £45, £60, £90, £150, £250
The attributes and their levels were defined in the survey (Appendix Questionnaire).

### Experimental Design

**Figure 1.**
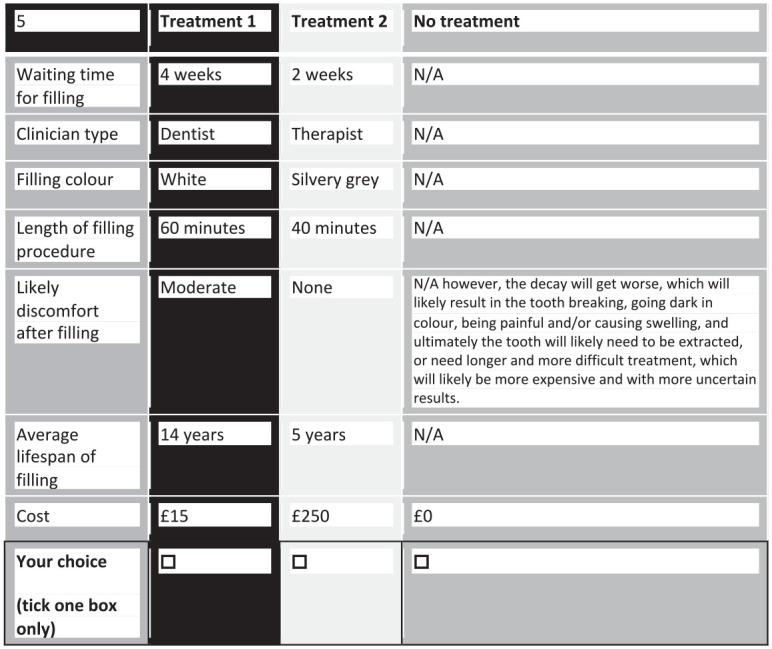
Example choice task.

There were 8,192 potential combinations of attribute levels, with none deemed totally implausible, and over 33 million choice sets. A fractional factorial D-optimal design was created using Ngene software (ChoiceMetrics). Based on a main effects full-profile approach, 64 choice questions were selected and split into 4 blocks of 16 questions (1 block only per respondent) (Bech et al. 2011). The model selection software was run overnight and the last 3 designs checked for within-block-level balance and appraised by their Pearson product moments to select the most appropriate design (Ngene design code, Appendix Fig. 1). Each choice question included 2 different treatment options and an opt-out (no treatment) to increase task realism. An example choice task is shown (Fig. 1). A repeated choice task was added to assess respondent consistency. A task in each block was selected where 1 choice appeared dominant (in that the levels were deemed better in all attributes), or close to dominant, to assess respondent rationality. Those failing the tests were not excluded from analysis based on expert guidance (Lancsar and Louviere 2006).

### Questionnaire Design

A cross-sectional online survey was developed (Appendix Questionnaire). The survey briefly explained the study and its purpose before confirming consent to participate. Demographic information and respondents’ experience of restorations were included, alongside the Modified Dental Anxiety Scale (Humphris et al. 1995). The survey also asked about attitudes toward restorative treatment and their perceived future need. It then explained the choice questions before presenting 17 choice tasks, alongside explanatory information. Piloting and think-aloud techniques (Coast et al. 2012) were used with dental and economic experts and the general public to assess the survey design, alongside a usability assessment with mobile devices.

### Sample

Sample size calculations for DCEs are imprecise (Bridges et al. 2011; Johnson et al. 2013). Guidance suggests a minimum subgroup sample size of 200 (Bridges et al. 2011). Therefore, to achieve sufficiently sized income subgroups, a sample size of 1,000 was deemed appropriate (Office for National Statistics [ONS] 2021). The DCE was distributed by Dynata using the FocusVision Decipher platform and their in-house sampling software to a representative online panel sample of the adult UK population based on population census data to obtain quotas on gender, age, and geographical region. Respondents received a small financial incentive for completing the survey. The data were electronically captured by Dynata in May/June 2021.

### Data Analysis

Data were analyzed using Stata software (version 17; StataCorp LP). Collinearity was assessed using variance inflation factors. Categorical variables were effects coded, and potentially continuous variables (waiting time, length of procedure, life span, and cost) were also explored categorically to assess assumptions of linearity using a conditional logit model. The utility function is shown and explained in Appendix Figure 2. Reference levels and their confidence intervals were calculated using the Delta method (Wooldridge 2002). Subgroups were defined as low (≤£20,000) or higher gross household income. Mixed logit models (McFadden and Train 2000) were explored with parameters modeled as fixed or random to assess intrasample preference heterogeneity and potentially continuous variables modeled as continuous or categorical where assumptions of linearity were questionable. Backward stepwise regressions were then carried out, changing the variable modeled as random with the highest standard deviation (SD) *P* value to nonrandom. Models were selected by highest log-likelihood. RAI and mWTP values were calculated (Mühlbacher and Johnson 2016).

## Results

In total, 1,002 respondents completed the survey. Internal validity was good, with 83% passing the consistency test and 91% passing the dominance test. Only 2% of respondents chose the opt-out for every question, and 1% always chose the same treatment option.

Demographic, dental experience, and attitudinal data are shown in Table 1. The sample shows similar proportions to the UK population in terms of gender, age, index of multiple deprivation (describing socioeconomic deprivation), and geographical location (ONS 2016). Comparison of reported gross household income with UK general population data is difficult because of available data presentation (decile means) (ONS 2021), but there is a broadly similar distribution of income. Based on 2009 Adult Dental Health Survey (ADHS 2009) data, the sample is representative of those with experience of a filling (85%), but edentulous respondents are slightly underrepresented.

**Table 1.** Demographic, Dental Experience, and Attitudinal Data of Respondents Including Income Subgroups.

Characteristic	Sample (*n* = 1,002)	Low Income (*n* = 221)	Higher Income (*n* = 727)
Age, mean (SD), y	48 (16)	49 (18)	47 (16)
Gender			
Female	50	57	48
Male	49	41	52
Other	<1	1	<1
PNTS	<1	0	<1
Residence			
England	81	80	82
Wales	5	8	4
Scotland	8	7	8
Northern Ireland	6	6	6
Index of multiple deprivation (deciles) (n = 986)			
1	11	16	9
2	10	15	9
3	10	7	11
4	10	14	9
5	9	12	8
6	11	10	11
7	11	11	11
8	10	5	11
9	9	6	10
10	10	4	11
Annual gross household income			
<£10,000	7	30	0
£10,000–£19,999	15	70	0
£20,000–£29,999	18	0	25
£30,000–£39,999	16	0	22
£40,000–£49,999	11	0	15
£50,000–£59,999	7	0	9
£60,000–£69,999	6	0	8
£70,000–£79,999	4	0	6
£80,000–£89,999	2	0	3
£90,000–£99,999	3	0	4
>£100,000	5	0	7
PNTS	5	0	0
Working status			
Employed (full-time or part-time)	56	32	65
Self-employed	7	6	6
Unemployed	5	12	2
Retired	22	29	19
Looking after home/family	4	6	3
Student	4	7	3
Other	3	7	1
Educational attainment (highest)			
Postgraduate degree	13	7	15
Undergraduate degree	25	20	28
A/AS level/Vocational A/AS level or equivalent	30	28	31
GCSE/Vocational GCSE/O level or equivalent	24	35	21
Lower than GCSE or equivalent level	7	11	5
Own natural teeth			
Yes	98	97	99
Filling in back tooth			
Yes	85	82	86
Silver (amalgam filling)			
Yes	79	78	79
White filling			
Yes	57	49	60
Environmental concern over filling materials (*n* = 911)			
Low	46	48	45
Medium	45	46	45
High	10	7	11
How at risk of needing a filling in future			
Low	29	29	28
Medium	51	51	52
High	20	20	20
Keeping my teeth is			
Important	87	87	87
Neither important nor unimportant	11	11	12
Unimportant	1	2	1
Highly anxious (Modified Dental Anxiety Scale)	26	28	25
Dental care provision			
NHS (pay)	52	50	53
NHS (exempt)	14	26	10
Insurance based	12	7	14
Private	16	12	17
Mixed NHS and private	6	5	6

Values are presented as percentages unless otherwise indicated.

GCSE, General Certificate of Secondary Education; NHS, National Health Service; PNTS, prefer not to say; SD, standard deviation.

Assumptions of linearity in the potentially continuous attributes were explored (Appendix Figs. 3–6). Model exploration resulted in a best-fit mixed logit specification with all parameters random and potentially continuous variables modeled continuously and linearly except waiting time, which was modeled categorically.

The results of the choice analysis are shown in Table 2 and Appendix Figure 7. The mean β values express the strength of respondent preferences relative to the mean, which is zero for categorical variables, and the strength of preference per unit change for continuous variables. All attributes exhibited some preference heterogeneity, as shown by significant SD *P* values, with the exception of clinician type. Overall, respondents were willing to pay £39.52 to reduce a 6-wk wait for treatment to 2 wk, £13.55 to have treatment by a dentist rather than a therapist, £41.66 to change filling color from silvery/gray to white, £0.27 per minute of reduced treatment time, £116.52 to move from persistent to no postoperative pain, and £5.44 per year of increased restoration longevity. An example of how the mWTP values could be used to comparatively value different restorations with different attribute levels is shown in Table 3.

**Table 2.** Mixed Logit Model Results Showing Main Effects Preferences and Willingness to Pay for Restoration Attributes.

		β Coefficient		mWTP (£)	
Attribute	Level	Mean	SD	Mean	95% CI
Waiting time for filling (wk)	0^a^	−0.020	—	−2.33	−11.90 to 7.24
	2	0.167*	0.004	19.40	8.52 to 30.28
	4	0.026	0.180*	3.05	−7.87 to 13.98
	6	−0.173*	0.004	−20.12	−32.17 to −8.08
Clinician	Dentist^a^	0.058*	—	6.77	−9.37 to −4.18
	Therapist	−0.058*	0.036	−6.77	4.18 to 9.37
Color	Silvery gray^a^	−0.179*	—	−20.83	−24.69 to 16.97
	White	0.179*	0.336*	20.83	16.97 to 24.69
Treatment time^b^	Per minute	−0.002*	0.000	−0.27	−0.40 to −0.15
Likely discomfort	None^a^	0.400*	—	46.46	38.89 to 54.04
	Mild	0.374*	0.030	43.36	36.99 to 49.74
	Moderate	−0.170*	0.0111	−19.78	−25.52 to −14.03
	Persistent	−0.603*	0.735*	−70.05	−79.70 to −60.40
Average life span^b^	Per year	0.047*	0.037*	5.44	4.49 to 6.38
Cost^b^	Per pound	−0.009*	0.010*	—	—
ASC	Treatment	4.257*	3.319*	494.26	421.63 to 566.89
	No treatment^a^	−4.257*	—	−494.26	−566.89 to −421.63

mWTP = −(β attribute^b^ or level/β cost); mWTP estimates are interpreted as the UK general population’s mean valuation of attributes and levels. Differences between mWTP values indicate how much the UK general population values moving from one level to another, or for a change of 1 unit.^b^ Therefore, moving from a waiting time of 0 wk to 2 wk would be valued = (19.40) – (−2.33) = £21.73; moving from a treatment time of 30 min to20 min would be valued (20 × −0.27) – (30 × −0.27) = £2.70. Respondents = 1,002; observations = 48,096; log-likelihood = −9,948.44; Akaike information criterion = 19,920.89; Bayesian information criterion = 20,026.26.

ASC, alternative specific constant; CI, confidence interval; mWTP, marginal willingness to pay; SD, standard deviation.

a Categorical reference level (in effects coded model).

b Continuously modeled attribute.

* *P* ≤ 0.001.

**Table 3.** Marginal Willingness to Pay for 2 Hypothetical Dental Restorations with Different Attribute Levels and Between Income Subgroups.

	Restoration 1 (composite, dentist)				Restoration 2 (amalgam, therapist)			
		mWTP (£)				mWTP (£)		
Attribute	Level	Full Sample	Low Income	Higher Income	Level	Full Sample	Low Income	Higher Income
Wait	4 wk	3.05	−3.52	3.95	2 wk	19.40	22.51	18.24
Clinician	Dentist	6.77	5.15	7.39	Therapist	−6.77	−5.15	−7.39
Color	White	20.83	8.90	25.15	Silvery gray	−20.83	−8.90	−25.15
Treatment time	34 min	−9.32	−10.98	−8.30	24 min	−6.56	−7.75	−5.86
Discomfort	Moderate	−19.78	−14.91	−21.46	Mild	43.36	34.27	46.89
Life span	7.98 y	43.39	24.77	50.71	11.05 y	60.09	34.30	70.21
Total		44.94	9.41	57.44		88.69	69.28	96.94

The mWTP values for attribute levels of any given restoration can be added to estimate its mean marginal value to the UK population. This allows calculation of WTP differences between restorations with different attribute levels (as shown here), which can then be used in economic evaluations. Treatment opt-in values were as follows: full sample, £539.20; low income, £324.74; higher income, £562.95.

mWTP, marginal willingness to pay.

Subgroup analysis based on income (Appendix Table 1) showed that on average, higher-income respondents value restoration longevity more than double (mWTP difference of £3.25/y) and a white restoration almost 3 times more than those with low income (mWTP difference of £16.25), and these differences were statistically significant. Higher-income respondents were, on average, willing to pay more to have treatment by a dentist rather than a therapist, to avoid postoperative discomfort and to avoid a 6-wk wait for a filling, although these differences were not statistically significant.

On average, low-income respondents valued shorter treatment times, willing to pay a third more per minute avoided, and also had a higher mean WTP for a 2-wk wait for a filling alongside lower mean WTP values for 0- or 4-wk waits than higher-income respondents, although these differences were not statistically significant.

The alternative specific constant (ASC) was large and positive, indicating that respondents much preferred treatment to no treatment, and ASC mWTP was significantly higher for the higher-income than the low-income group.

**Figure 2.**
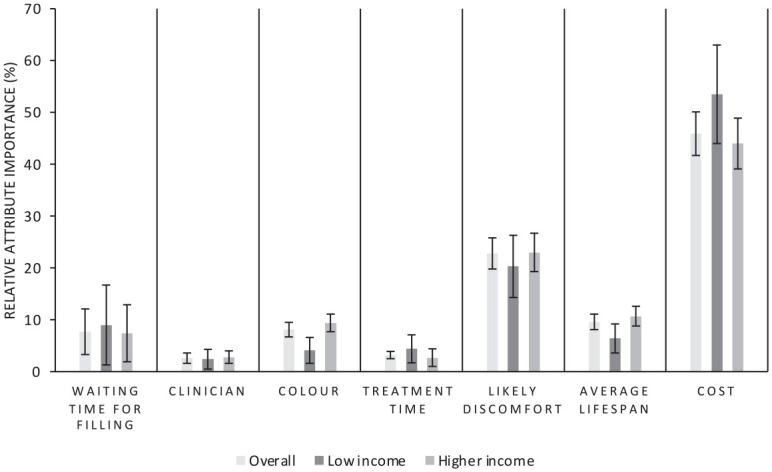
storation relative attribute importance: overall UK population and by income.

RAI is presented in Figure 2, based on Appendix Table 2, which shows the proportionate valuation of each restoration attribute based on the range of valuation of levels within each attribute. This showed that cost is the most important attribute for the general public when selecting a posterior dental restoration, being 2.0 times more important than the next most important attribute, which was likely discomfort after the filling. Discomfort, in turn, was 2.4 times more important than average life span, with color and waiting time next most important, but these 3 attributes were not statistically significantly different from each other. Treatment time and clinician type, which again were not statistically significantly different from one another, were the least important attributes. When analyzed by income groups, RAI changed, resulting in different ordering in the importance of life span, color, waiting time, treatment time, and clinician, but with only color statistically significantly different between groups.

## Discussion

This DCE is the first to explore general population preferences for direct posterior dental restorations. Overall, all attributes were valued by the respondents, and the valuation of the levels within each attribute was generally as expected (i.e., increased restoration longevity resulted in higher valuations). This shows that respondents were trading across all included attributes and were able to discriminate between the levels presented in the choice tasks, providing justification for the design and confidence in the results.

The mWTP values obtained can be used to value different direct posterior restorations, which have attribute levels included in the DCE, as shown in Table 3, which in turn can be used to broaden the scope of economic evaluations, especially when used to value the interventions in cost-benefit analyses. Nearly all previous economic evaluations of posterior restorations focus on restoration or tooth longevity as the primary outcome measure. This is far from being the most important attribute when judged by the general public, with cost and likely discomfort after filling having much higher RAI. Longevity also has a markedly lower RAI in the low-income group. This suggests that these previous economic analyses are excessively narrow in their scope. Valuing restorations by adding mWTP estimates of their attributes takes a broader, patient-centered approach. It also shows how preferences differ with income. This information is critical for policy-makers to consider when redesigning restorative dental services, which is pertinent given the recent move toward amalgam phase-down. This article highlights the potential for health economics to move beyond limited cost-effectiveness analyses, by combining innovative approaches and considering multiple perspectives to address complex decision problems in oral health and care (Listl et al. 2019; Listl et al. 2022).

Respondents valued treatment over no treatment, although differences existed in income groups, as they did in attribute levels. Compared to higher-income respondents, restoration longevity was of much lower importance to low-income respondents, although waiting time and treatment time were of higher importance, with increased mWTP values. This could be due to a reduced willingness to wait, or to sacrifice time in the short term with an increased discounting of future benefits. This in turn could be caused by a wish to minimize time off work and a reduced ability to pay, or simply a preference for short-term versus long-term benefits. Likewise, restoration color was much less important to the low-income group, which suggests they value appearance less. Despite these data on sample means, significant variation exists between individuals within the sample and within the subgroups in all attributes except clinician type.

Respondents favored shorter waiting times, with an optimal wait of 2 wk, but did not discriminate hugely between waiting times of 4 wk and under. There was a significant drop in valuation for a 6-wk wait, however, which was more marked in the low-income group, which has implications when planning dental service provision. Given the increased time taken and cost to place composite restorations and their reduced longevity compared to amalgam (Bailey et al. 2022a; Khangura et al. 2018), the amalgam phase-down and potential phase-out will likely mean that clinicians will have more restorative work to do. This potentially means increased waiting times to access care with current workforce levels (Bailey et al. 2022b). These issues potentially affect those of low income by limiting the service characteristics that they desire and could reduce their access to and uptake of care. This is countered by the higher value placed on having a white compared to a silvery-gray filling. This value was significantly reduced, however, in the low-income group. An amalgam phase-out therefore potentially risks exacerbating already existing socioeconomic disparities in oral health (Steele et al. 2015), but an economic evaluation is required to better understand the potential impacts.

The general public prefers to have their restorations placed by dentists rather than therapists. It is important to consider if the preference (valued as the difference in mWTP) is offset by the increased cost associated with the dentist performing the filling and the other patient-centered outcomes obtained by differing clinician types (which may not be the same; Bailey et al. 2022a). Care responsibilities within the dental team, policy decisions, and workforce planning can then be considered rationally, weighing up costs and benefits of alternatives to optimize the use of scarce resources across a diverse population (Listl et al. 2019).

Preferences for attributes of a posterior direct restoration differ between patients and clinicians (Espelid et al. 2006), and this research shows that interindividual preferences vary in the UK general population. Clinicians should therefore not make assumptions about what individual patients value. The attributes assessed were all of importance to the general public in aggregate. How they vary between the available direct posterior restorative options should therefore be discussed with individual patients when obtaining consent.

Ability to pay often affects willingness to pay (Tan et al. 2017), which was again replicated in this study. It is therefore important to consider how this might affect choices among those with low income when making policy decisions. Although this sample was generally representative of the UK general population on many levels, there was a higher proportion of highly anxious respondents, which could have affected the results. There are also potential confounding factors in splitting the sample, as educational attainment, for example, also varies between subgroups. Respondents’ previous dental experiences and potential varied interpretation of no treatment as delaying care could affect the results, as could the absence of “unknown” options when asking for descriptive data.

Hypothetical bias was mitigated against by using PPI in the development and design stages. Where respondents have experience of the treatments being valued, as the vast majority did in this study, it is also likely to be minimized. There are, however, limited numbers of studies investigating the ability of stated preference techniques to predict revealed preferences in dentistry, with equivocal results (Vernazza et al. 2015).

## Conclusions

The UK general population values direct posterior restorations highly, placing importance on a variety of restoration attributes beyond longevity. These include process of care, such as waiting time for a filling and treatment time, as well as aesthetics, the care provider, postoperative complications, and, most important, cost. Clinicians should understand potential drivers of restoration choice, so they can be discussed with individual patients to obtain consent. When contemplating the potential phase-out of amalgam restorations, policy-makers should consider general population preferences for services and outcomes, with an awareness that income affects these.

## Author Contributions

O. Bailey, contributed to conception, design, data acquisition, analysis, and interpretation, drafted and critically revised the manuscript; S. Stone, contributed to conception, design, and data interpretation, critically revised the manuscript; L. Ternent, contributed to conception, design, data analysis, and interpretation, critically revised the manuscript; C.R. Vernazza, contributed to conception, design, data acquisition, and interpretation, critically revised the manuscript. All authors gave final approval and agree to be accountable for all aspects of the work.

## Supplemental Material

sj-docx-1-jdr-10.1177_00220345221108699 – Supplemental material for Public Valuation of Direct Restorations: A Discrete Choice Experiment Click here for additional data file. Supplemental material, sj-docx-1-jdr-10.1177_00220345221108699 for Public Valuation of Direct Restorations: A Discrete Choice Experiment by O. Bailey, S. Stone, L. Ternent and C.R. Vernazza in Journal of Dental Research
