# Supplementary material for: Public Valuation of Direct Restorations: A Discrete Choice Experiment
Source: J Dent Res. 2022 Jul 25;101(13):1572–9. doi: 10.1177/00220345221108699 (PMC9693719; doi:10.1177/00220345221108699)
Supplement: sj-docx-1-jdr-10.1177_00220345221108699 – Supplemental material for Public Valuation of Direct Restorations: A Discrete Choice Experiment [file sj-docx-1-jdr-10.1177_00220345221108699.docx]

**Public Valuation of Direct Restorations: A Discrete Choice Experiment**

Bailey O, Stone S, Ternent L, Vernazza C

**Appendix**

| **Contents** | **Page(s)** |
| --- | --- |
| Questionnaire | 2-37 |
| Appendix Figure 1. Ngene design code | 38 |
| Appendix Figure 2. Utility function | 38 |
| Appendix Figure 3. General public valuation of varying wait for filling | 39 |
| Appendix Figure 4. General public valuation of varying treatment time for filling | 39 |
| Appendix Figure 5. General public valuation of varying lifespan of filling | 39 |
| Appendix Figure 6. General public valuation of varying cost of filling | 40 |
| Appendix Figure 7. Overall UK population marginal willingness to pay for direct posterior restoration attributes | 40 |
| Appendix Table 1. Mixed logit model results showing preferences and willingness to pay for restoration attributes by income sub-groups | 41 |
| Appendix Table 2. Relative attribute importance: overall and by income | 41 |

**Questionnaire**

Survey presented as a word document. It was reformatted onto the Decipher platform before being sent electronically by the distribution company (Dynata) using their in-house sampling tool. This is Block 1. There were four blocks. The blocks differ only in the choice questions.

Introduction

This survey is about dental fillings and how you value different aspects of them.

You will be presented with two different imaginary situations of having a filling in a tooth, with the likely outcomes, and you will be asked to choose between them.

This will be explained in more detail if you wish to take part.

The results of the survey will help decision makers to take patients’ opinions into account and therefore make better decisions, when deciding how to provide dental fillings in the UK.

The questionnaire should take around 10 minutes to complete.

Information (and GDPR statement)

Information which could identify you will be separated from responses before they are transferred and analysed at Newcastle University, therefore all information will be pseudonymous.

This study has ethical approval from Newcastle University**.**

*Newcastle University will be using information from you in order to undertake this research study. Dynata will act as the data controller for this study. This means that Dynata and Newcastle University are responsible for looking after your information and using it properly. When we use personally-identifiable information from people who have agreed to take part in research, we ensure that it is in the public interest.*

*Dynata will use your name and email address to contact you about the research study. They will receive your responses should you choose to take part in the study. This information will be pseudonymised before being transferred to Newcastle University and will not be combined with other information in a way that could identify you. The information will only be used for the purpose research, and cannot be used to contact you. It will not be used to make decisions about future services available to you. Your rights to access, change or move your information are limited, as Newcastle University needs to manage your information in specific ways in order for the research to be reliable and accurate. If you withdraw from the study, Newcastle University will keep the information about you that has already been obtained. To safeguard your rights, the minimum personally-identifiable information will be used. You can find out more about how Newcastle University uses your information at* [***https://www.ncl.ac.uk/data.protection/dataprotectionpolicy/privacynotice/***](https://www.ncl.ac.uk/data.protection/dataprotectionpolicy/privacynotice/) *and/or by contacting Newcastle University’s Data Protection Officer (Maureen Wilkinson,* [***rec-man@ncl.ac.uk***](mailto:rec-man@ncl.ac.uk)*).*

*Newcastle University will not have access to your name or email address, but we will use your post code and other personal information provided by you in order to ascertain how representative the sample is of the general population, and to assess how or if this information affects the results of the research. Individuals at Newcastle University may look at your research data to check the accuracy of the research study. The only individuals at Newcastle University who will have access to information that identifies you will be individuals who are performing the research, or auditing the data collection process.*

*If you agree to take part in the research study, information provided by you may be shared with researchers running other research studies at Newcastle University. Your information will only be used by Newcastle University and researchers to conduct research.*

Consent

1. I confirm that I have read and understood the purpose of this research and have had the opportunity to consider the information and my involvement.

• Yes

• No

1. I understand that my involvement is voluntary and I consent to participate in this study.

• Yes

• No

Respondent information

The following questions ask about your characteristics so that we can demonstrate that we have collected information from a representative sample of people living in the UK.

It will also allow us to explore how people’s varying characteristics affect their choices for dental fillings and therefore potentially provide solutions that will be acceptable to people with different characteristics.

Age

S1. What is your age in years?

Gender

S2. What gender are you?

- Female
- Male
- Other
- Prefer not to say

S3. Which region do you live in?

North East

North West

Yorkshire and the Humber

East Midlands

West Midlands

East of England

London

South East

South West

Wales

Scotland

Northern Ireland

Postcode

S4. Please enter your home postcode

Education

S5. Move down the list and tick your highest level of educational qualification

• Postgraduate degree
• Undergraduate degree
• Higher qualification below degree level
• A-level/Vocational A-level or equivalent
• AS-level/Vocational AS-level or equivalent
• International baccalaureate
• O-levels or equivalent
• GCSE/Vocational GCSE or equivalent
• Other work related or professional qualification • School Leavers Certificate
• None

Working status

S6. Which of the following best describes your current working status?

- Working
  • Employed (full-time or part-time)

• Self-employed

- Unemployed
- Retired
- Student
- Apprentice
- Furloughed
- Maternity leave
- Short-term sick leave
- Long-term sick leave
- Looking after home/family

Annual household income

S7. Please provide an estimate of your combined annual gross household income last year, before taxes and deductions Single code

Up to £10,000

£10,000 - £19,999

£20,000 - £29,999

£30,000 - £39,999

£40,000 - £49,999

£50,000 - £59,999

£60,000 - £69,999

£70,000 - £79,999

£80,000 - £89,999

£90,000 - £99,999

£100,000 or more

Teeth

First, we’d like to know a little about your teeth, and how your dental care is provided

Please answer the following questions:

Teeth

1. Do you have any of your own natural teeth in your mouth?

• Yes

• No

1. Have you ever had a filling in a back tooth? Teeth behind line on picture classed as back teeth (same for upper teeth). This does not include a crown or onlay (‘cap’) that was made outside of your mouth and needed an impression or a scan of your teeth.

• Yes

• No


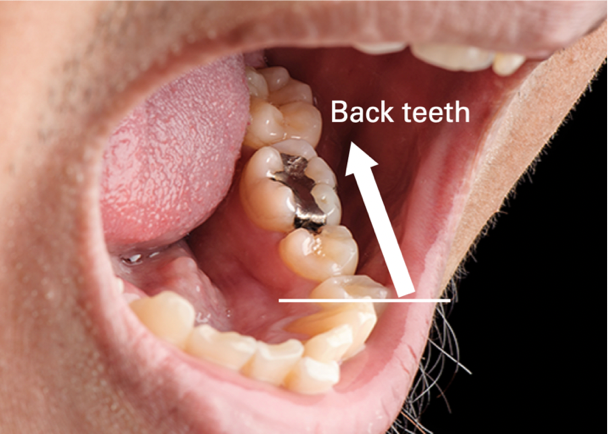


Teeth continued (dependent on previous question)

1. Have you ever had a silver (amalgam) filling in a back tooth?

• Yes
• No

1. Have you ever had a white filling in a back tooth?

• Yes
• No

1. What is your level of concern about the environmental impact of dental filling materials?

- Low
- Medium
- High

Risk

1. How at risk would you say you are of needing a filling in one of your back teeth in the future?

- Low
- Medium
- High

Importance of your teeth

1. I feel that keeping my natural teeth is:

• Important
• Neither important nor unimportant

• Unimportant

Dental anxiety

**Can you tell us how anxious, if at all, you get when visiting the dentist?**

1. If you went to your dentist for TREATMENT TOMORROW, how would you feel?

Not anxious

Slightly anxious

Fairly anxious

Very anxious

Extremely anxious

1. If you were sitting in the WAITING ROOM (waiting for treatment), how would you feel?

Not anxious

Slightly anxious

Fairly anxious

Very anxious

Extremely anxious

1. If you were about to have a TOOTH DRILLED, how would you feel?

Not anxious

Slightly anxious

Fairly anxious

Very anxious

Extremely anxious

1. If you were about to have your TEETH SCALED AND POLISHED, how would you feel?

Not anxious

Slightly anxious

Fairly anxious

Very anxious

Extremely anxious

1. If you were about to have a LOCAL ANAESTHETIC INJECTION in your gum, above an upper back molar tooth, how would you feel?

Not anxious

Slightly anxious

Fairly anxious

Very anxious

Extremely anxious

15.Please indicate how your dental care is provided

- NHS (you pay the NHS ‘band’ charges)
- NHS (you do not pay and are exempt from NHS charges)
- Insurance based, you pay a monthly fee – this includes all treatment except laboratory bills
- Insurance based, you pay a monthly fee with discounts on any private treatment provided
- Privately (you pay full costs of private treatment)
- Mixed of some NHS banded and some private treatments

The choice questions

In the questions which appear on the following pages, you will be presented with two imaginary treatments.

Each treatment describes a different imaginary situation of having a filling in a tooth, with the likely outcomes.

Please think about each option, as if you were making a decision between the two options in real life circumstances, and tell us which treatment you would choose.

If you do not have any teeth, obviously you will never require a filling, but try to imagine yourself with teeth and make the choice as you would if you had teeth, because we are really interested to hear your opinions too.


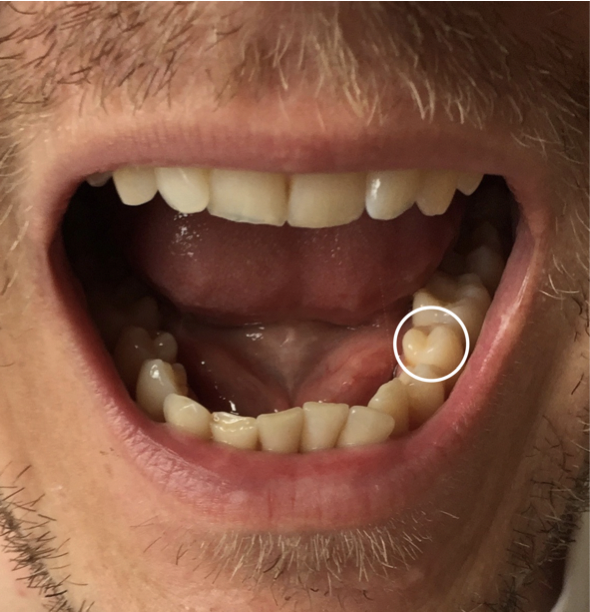

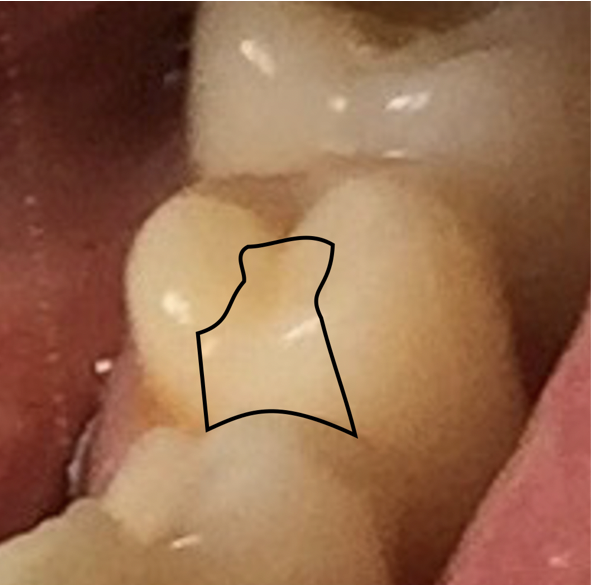


A filling is placed when a tooth is decayed.

Imagine the tooth circled is decayed, but not causing you pain, and needs a filling with the outline shown on the close-up view of the tooth.

Assume that the clinician providing the treatment gives a detailed explanation of the procedure and has a caring and friendly manner.

There is also another choice available to you- you can choose not to have any treatment done. If you choose the option ‘no treatment’, this means that the decay will get worse, which will likely result in the tooth:

breaking
going dark in colour
becoming painful and/or infected which may cause a swelling or an abscess

Ultimately the tooth will likely need to be extracted, or need longer and more difficult root canal treatment. This more difficult treatment will likely be more expensive and with more uncertain results.

Also imagine that you cannot shop around and get a different price for the required treatment somewhere else. For each choice question, imagine that the treatments would be exactly the same at any dental practice you went to (including the prices).

Each treatment you will be presented with relates to the above situation, and includes seven different aspects of having the filling, covering the following areas:

**Waiting time for filling**

The amount of time you have to wait to have the filling done in weeks- 0, 2, 4 or 6 weeks

**Clinician type**

Dentist or dental therapist.
The key differences between a dentist and dental therapist are:

Therapists:

- can do simple fillings, scaling and deep cleaning, but not more complicated procedures like

crowns, root canal treatments or replacing missing teeth, which are performed by dentists.

- can provide simple fillings direct to patients, or under the guidance of a dentist
- Are registered dental professionals required to study at university for two to four years to gain a diploma or degree, rather than five years for a dentist to gain their degree.

**Filling colour**White **Or** Silvery grey

**
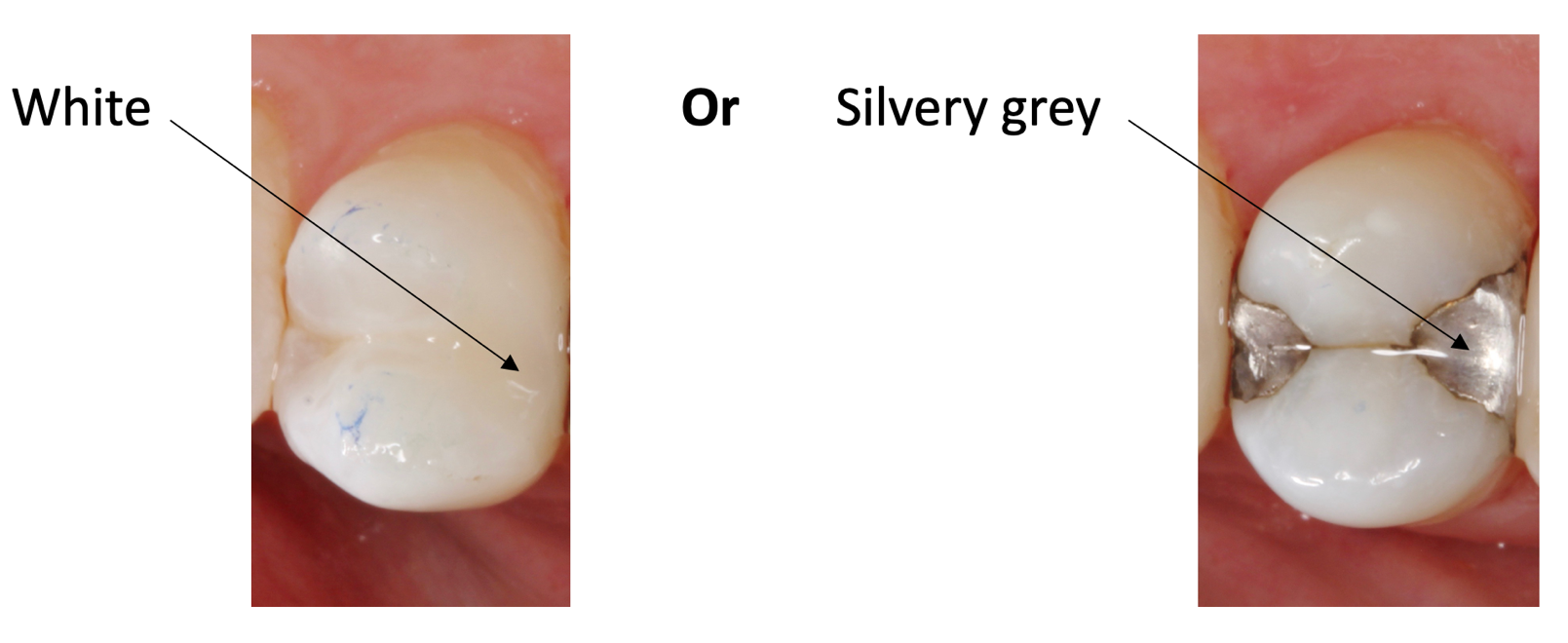
**

**Length of filling procedure**

This is how long you will need to be in the dental chair to have the filling placed in minutes- 20, 40, 60 or 80 minutes. (You should **not** consider that the quality of the filling will increase with increased time or vice- versa.)

**Likely discomfort after filling**

This relates to the likely level of discomfort when eating and drinking after having a filling placed.
This could be:
None
Mild (short-lived low-level sensitivity for 2-4 weeks not causing problems with function)
Moderate (requiring painkillers and may mean that you would avoid eating, chewing or drinking certain foods or drinks for 2-4 weeks)

Persistent (requiring reattendance at the dental practice for the management of a problem after 2-4 weeks)

**Filling will last on average**

The likely average time in years that the filling lasts until it needs another procedure- for example, until it needs a replacement filling- 5, 8, 11, 14 years

**Cost**

The out of pocket fee in UK pounds sterling which you would have to pay for the filling in each scenario- £15, £25, £35, £45, £60, £90, £150, £250

Going to an appointment for treatment may also mean that you have transport costs and will miss work, or other activities that you do, which could affect your disposable income, wage and leisure time.

It is likely that once a filling has been placed, it will need to be replaced and each time it is replaced there will be associated costs involved with this. Each time a filling is replaced, the filling is also likely to get bigger, which could impact on the need for more complicated future treatments, which may be more expensive, and ultimately could reduce the amount of time the tooth will last in your mouth before needing extraction. Replacing the missing tooth, would also have costs.

Please factor these things in when choosing.

We are interested in your choices of filling procedures and outcomes.

You may think the choice between different scenarios seems repetitive and irrelevant, but your answers, in combination with responses from other people, will help decision makers to make more informed, patient- centred decisions when deciding how to provide dental fillings in the UK.

It is important that you consider your choices carefully. There are no right or wrong answers; it is your personal choice that is important.

Choice questions

| 5 | **Treatment 1** | **Treatment 2** | **No treatment** |
| --- | --- | --- | --- |
| Waiting time for filling | 4 weeks | 2 weeks | N/A |
| Clinician type | Dentist | Therapist | N/A |
| Filling colour | White | Silvery grey | N/A |
| Length of filling procedure | 60 minutes | 40 minutes | N/A |
| Likely discomfort after filling | Moderate | None | N/A however, the decay will get worse, which will likely result in the tooth breaking, going dark in colour, being painful and/or causing swelling, and ultimately the tooth will likely need to be extracted, or need longer and more difficult treatment, which will likely be more expensive and with more uncertain results. |
| Average lifespan of filling | 14 years | 5 years | N/A |
| Cost | £15 | £250 | £0 |
| **Your choice**  **(tick one box only)** | **☐** | **☐** | **☐** |

| 12 | **Treatment 1** | **Treatment 2** | **No treatment** |
| --- | --- | --- | --- |
| Waiting time for filling | 4 weeks | 2 weeks | N/A |
| Clinician type | Therapist | Dentist | N/A |
| Filling colour | Silvery grey | White | N/A |
| Length of filling procedure | 40 minutes | 60 minutes | N/A |
| Likely discomfort after filling | None | Persistent | N/A however, the decay will get worse, which will likely result in the tooth breaking, going dark in colour, being painful and/or causing swelling, and ultimately the tooth will likely need to be extracted, or need longer and more difficult treatment, which will likely be more expensive and with more uncertain results. |
| Average lifespan of filling | 5 years | 14 years | N/A |
| Cost | £250 | £15 | £0 |
| Your choice  (tick one box only) | **☐** | **☐** | **☐** |

| 16 | **Treatment 1** | **Treatment 2** | **No treatment** |
| --- | --- | --- | --- |
| Waiting time for filling | 6 weeks | 0 weeks | N/A |
| Clinician type | Dentist | Therapist | N/A |
| Filling colour | Silvery grey | White | N/A |
| Length of filling procedure | 40 minutes | 60 minutes | N/A |
| Likely discomfort after filling | Moderate | Mild | N/A however, the decay will get worse, which will likely result in the tooth breaking, going dark in colour, being painful and/or causing swelling, and ultimately the tooth will likely need to be extracted, or need longer and more difficult treatment, which will likely be more expensive and with more uncertain results. |
| Average lifespan of filling | 8 years | 11 years | N/A |
| Cost | £60 | £45 | £0 |
| **Your choice**  **(tick one box only)** | **☐** | **☐** | **☐** |

| 23 | **Treatment 1** | **Treatment 2** | **No treatment** |
| --- | --- | --- | --- |
| Waiting time for filling | 6 weeks | 0 weeks | N/A |
| Clinician type | Therapist | Dentist | N/A |
| Filling colour | Silvery grey | White | N/A |
| Length of filling procedure | 80 minutes | 20 minutes | N/A |
| Likely discomfort after filling | Persistent | None | N/A however, the decay will get worse, which will likely result in the tooth breaking, going dark in colour, being painful and/or causing swelling, and ultimately the tooth will likely need to be extracted, or need longer and more difficult treatment, which will likely be more expensive and with more uncertain results. |
| Average lifespan of filling | 11 years | 8 years | N/A |
| Cost | £35 | £90 | £0 |
| **Your choice**  **(tick one box only)** | **☐** | **☐** | **☐** |

| 26 | **Treatment 1** | **Treatment 2** | **No treatment** |
| --- | --- | --- | --- |
| Waiting time for filling | 0 weeks | 6 weeks | N/A |
| Clinician type | Dentist | Therapist | N/A |
| Filling colour | White | Silvery grey | N/A |
| Length of filling procedure | 20 minutes | 80 minutes | N/A |
| Likely discomfort after filling | Persistent | None | N/A however, the decay will get worse, which will likely result in the tooth breaking, going dark in colour, being painful and/or causing swelling, and ultimately the tooth will likely need to be extracted, or need longer and more difficult treatment, which will likely be more expensive and with more uncertain results. |
| Average lifespan of filling | 5 years | 14 years | N/A |
| Cost | £150 | £25 | £0 |
| **Your choice**  **(tick one box only)** | **☐** | **☐** | **☐** |

| 27 | **Treatment 1** | **Treatment 2** | **No treatment** |
| --- | --- | --- | --- |
| Waiting time for filling | 4 weeks | 2 weeks | N/A |
| Clinician type | Therapist | Dentist | N/A |
| Filling colour | White | Silvery grey | N/A |
| Length of filling procedure | 60 minutes | 40 minutes | N/A |
| Likely discomfort after filling | Moderate | Mild | N/A however, the decay will get worse, which will likely result in the tooth breaking, going dark in colour, being painful and/or causing swelling, and ultimately the tooth will likely need to be extracted, or need longer and more difficult treatment, which will likely be more expensive and with more uncertain results. |
| Average lifespan of filling | 8 years | 11 years | N/A |
| Cost | £60 | £45 | £0 |
| **Your choice**  **(tick one box only)** | **☐** | **☐** | **☐** |

| 31 | **Treatment 1** | **Treatment 2** | **No treatment** |
| --- | --- | --- | --- |
| Waiting time for filling | 4 weeks | 2 weeks | N/A |
| Clinician type | Dentist | Therapist | N/A |
| Filling colour | Silvery grey | White | N/A |
| Length of filling procedure | 80 minutes | 20 minutes | N/A |
| Likely discomfort after filling | Mild | None | N/A however, the decay will get worse, which will likely result in the tooth breaking, going dark in colour, being painful and/or causing swelling, and ultimately the tooth will likely need to be extracted, or need longer and more difficult treatment, which will likely be more expensive and with more uncertain results. |
| Average lifespan of filling | 8 years | 11 years | N/A |
| Cost | £45 | £60 | £0 |
| **Your choice**  **(tick one box only)** | **☐** | **☐** | **☐** |

| 33 | **Treatment 1** | **Treatment 2** | **No treatment** |
| --- | --- | --- | --- |
| Waiting time for filling | 0 weeks | 6 weeks | N/A |
| Clinician type | Therapist | Dentist | N/A |
| Filling colour | White | Silvery grey | N/A |
| Length of filling procedure | 40 minutes | 60 minutes | N/A |
| Likely discomfort after filling | Moderate | Persistent | N/A however, the decay will get worse, which will likely result in the tooth breaking, going dark in colour, being painful and/or causing swelling, and ultimately the tooth will likely need to be extracted, or need longer and more difficult treatment, which will likely be more expensive and with more uncertain results. |
| Average lifespan of filling | 11 years | 8 years | N/A |
| Cost | £35 | £90 | £0 |
| **Your choice**  **(tick one box only)** | **☐** | **☐** | **☐** |

| 23 | **Treatment 1** | **Treatment 2** | **No treatment** |
| --- | --- | --- | --- |
| Waiting time for filling | 0 weeks | 6 weeks | N/A |
| Clinician type | Dentist | Therapist | N/A |
| Filling colour | White | Silvery grey | N/A |
| Length of filling procedure | 20 minutes | 80 minutes | N/A |
| Likely discomfort after filling | None | Persistent | N/A however, the decay will get worse, which will likely result in the tooth breaking, going dark in colour, being painful and/or causing swelling, and ultimately the tooth will likely need to be extracted, or need longer and more difficult treatment, which will likely be more expensive and with more uncertain results. |
| Average lifespan of filling | 8 years | 11 years | N/A |
| Cost | £90 | £35 | £0 |
| **Your choice**  **(tick one box only)** | **☐** | **☐** | **☐** |

| 40 | **Treatment 1** | **Treatment 2** | **No treatment** |
| --- | --- | --- | --- |
| Waiting time for filling | 0 weeks | 6 weeks | N/A |
| Clinician type | Dentist | Therapist | N/A |
| Filling colour | White | Silvery grey | N/A |
| Length of filling procedure | 20 minutes | 80 minutes | N/A |
| Likely discomfort after filling | None | Mild | N/A however, the decay will get worse, which will likely result in the tooth breaking, going dark in colour, being painful and/or causing swelling, and ultimately the tooth will likely need to be extracted, or need longer and more difficult treatment, which will likely be more expensive and with more uncertain results. |
| Average lifespan of filling | 14 years | 5 years | N/A |
| Cost | £35 | £90 | £0 |
| **Your choice**  **(tick one box only)** | **☐** | **☐** | **☐** |

| 43 | **Treatment 1** | **Treatment 2** | **No treatment** |
| --- | --- | --- | --- |
| Waiting time for filling | 0 weeks | 6 weeks | N/A |
| Clinician type | Therapist | Dentist | N/A |
| Filling colour | White | Silvery grey | N/A |
| Length of filling procedure | 20 minutes | 80 minutes | N/A |
| Likely discomfort after filling | Persistent | None | N/A however, the decay will get worse, which will likely result in the tooth breaking, going dark in colour, being painful and/or causing swelling, and ultimately the tooth will likely need to be extracted, or need longer and more difficult treatment, which will likely be more expensive and with more uncertain results. |
| Average lifespan of filling | 5 years | 14 years | N/A |
| Cost | £90 | £35 | £0 |
| **Your choice**  **(tick one box only)** | **☐** | **☐** | **☐** |

| 49 | **Treatment 1** | **Treatment 2** | **No treatment** |
| --- | --- | --- | --- |
| Waiting time for filling | 2 weeks | 4 weeks | N/A |
| Clinician type | Dentist | Therapist | N/A |
| Filling colour | White | Silvery grey | N/A |
| Length of filling procedure | 80 minutes | 20 minutes | N/A |
| Likely discomfort after filling | Moderate | None | N/A however, the decay will get worse, which will likely result in the tooth breaking, going dark in colour, being painful and/or causing swelling, and ultimately the tooth will likely need to be extracted, or need longer and more difficult treatment, which will likely be more expensive and with more uncertain results. |
| Average lifespan of filling | 8 years | 11 years | N/A |
| Cost | £25 | £150 | £0 |
| **Your choice**  **(tick one box only)** | **☐** | **☐** | **☐** |

| 50 | **Treatment 1** | **Treatment 2** | **No treatment** |
| --- | --- | --- | --- |
| Waiting time for filling | 0 weeks | 6 weeks | N/A |
| Clinician type | Therapist | Dentist | N/A |
| Filling colour | Silvery grey | White | N/A |
| Length of filling procedure | 20 minutes | 80 minutes | N/A |
| Likely discomfort after filling | None | Persistent | N/A however, the decay will get worse, which will likely result in the tooth breaking, going dark in colour, being painful and/or causing swelling, and ultimately the tooth will likely need to be extracted, or need longer and more difficult treatment, which will likely be more expensive and with more uncertain results. |
| Average lifespan of filling | 14 years | 5 years | N/A |
| Cost | £25 | £150 | £0 |
| **Your choice**  **(tick one box only)** | **☐** | **☐** | **☐** |

| 52 | **Treatment 1** | **Treatment 2** | **No treatment** |
| --- | --- | --- | --- |
| Waiting time for filling | 6 weeks | 0 weeks | N/A |
| Clinician type | Dentist | Therapist | N/A |
| Filling colour | Silvery grey | White | N/A |
| Length of filling procedure | 40 minutes | 60 minutes | N/A |
| Likely discomfort after filling | Mild | Moderate | N/A however, the decay will get worse, which will likely result in the tooth breaking, going dark in colour, being painful and/or causing swelling, and ultimately the tooth will likely need to be extracted, or need longer and more difficult treatment, which will likely be more expensive and with more uncertain results. |
| Average lifespan of filling | 11 years | 8 years | N/A |
| Cost | £150 | £25 | £0 |
| **Your choice**  **(tick one box only)** | **☐** | **☐** | **☐** |

| 56 | **Treatment 1** | **Treatment 2** | **No treatment** |
| --- | --- | --- | --- |
| Waiting time for filling | 4 weeks | 2 weeks | N/A |
| Clinician type | Dentist | Therapist | N/A |
| Filling colour | Silvery grey | White | N/A |
| Length of filling procedure | 20 minutes | 80 minutes | N/A |
| Likely discomfort after filling | Mild | Persistent | N/A however, the decay will get worse, which will likely result in the tooth breaking, going dark in colour, being painful and/or causing swelling, and ultimately the tooth will likely need to be extracted, or need longer and more difficult treatment, which will likely be more expensive and with more uncertain results. |
| Average lifespan of filling | 14 years | 5 years | N/A |
| Cost | £150 | £25 | £0 |
| **Your choice**  **(tick one box only)** | **☐** | **☐** | **☐** |

| 58 | **Treatment 1** | **Treatment 2** | **No treatment** |
| --- | --- | --- | --- |
| Waiting time for filling | 2 weeks | 4 weeks | N/A |
| Clinician type | Therapist | Dentist | N/A |
| Filling colour | Silvery grey | White | N/A |
| Length of filling procedure | 60 minutes | 40 minutes | N/A |
| Likely discomfort after filling | Persistent | Mild | N/A however, the decay will get worse, which will likely result in the tooth breaking, going dark in colour, being painful and/or causing swelling, and ultimately the tooth will likely need to be extracted, or need longer and more difficult treatment, which will likely be more expensive and with more uncertain results. |
| Average lifespan of filling | 14 years | 5 years | N/A |
| Cost | £90 | £35 | £0 |
| **Your choice**  **(tick one box only)** | **☐** | **☐** | **☐** |

| 62 | **Treatment 1** | **Treatment 2** | **No treatment** |
| --- | --- | --- | --- |
| Waiting time for filling | 4 weeks | 2 weeks | N/A |
| Clinician type | Dentist | Therapist | N/A |
| Filling colour | White | Silvery grey | N/A |
| Length of filling procedure | 60 minutes | 40 minutes | N/A |
| Likely discomfort after filling | Mild | Persistent | N/A however, the decay will get worse, which will likely result in the tooth breaking, going dark in colour, being painful and/or causing swelling, and ultimately the tooth will likely need to be extracted, or need longer and more difficult treatment, which will likely be more expensive and with more uncertain results. |
| Average lifespan of filling | 11 years | 8 years | N/A |
| Cost | £250 | £15 | £0 |
| **Your choice**  **(tick one box only)** | **☐** | **☐** | **☐** |

Thank you for completing the survey!

**Appendix Figure 1**

Ngene software design code

This code generated a d-efficient design to estimate a mixed logit preference model

| ;alts = alt1, alt2, alt3 |
| --- |
| ;block = 4 |
| ;rows = 64 |
| ;eff = (mnl, d) |
| ;model: |
| U(alt1) = asc1 + b1 * WAIT[0,2,4,6] + b2.dummy[0] * CLINICIAN[1,2] + b3.dummy[0] * COLOUR [1,2] + b4 * TIME[20,40,60,80] + b5.dummy[0\|0\|0] * DISCOMFORT[1,2,3,4] + b6[(u,0,0.001)] * LIFESPAN[5,8,11,14] + b7[(u,-0.001,0)] * COST[25,50,75,100,150,200,250,300] / |
| U(alt2) = asc2 + b1 * WAIT + b2.dummy * CLINICIAN + b3.dummy * COLOUR + b4 * TIME + b5.dummy * DISCOMFORT + b6 * LIFESPAN + b7 * COST |
| $ |

**Appendix Figure 2**

Utility function

$$V_{j}=\alpha ASC+\beta_{1}{Wait}_{0}+\beta_{2}{Wait}_{2}+\beta_{3}{Wait}_{4}+\beta_{4}{Wait}_{6}+\beta_{5}{Clinician}_{Dentist}+\beta_{6}{Clinician}_{Therapist}+\beta_{7}{Colour}_{Silvery grey}+\beta_{8}{Colour}_{White}+\beta_{9}Length+\beta_{10}{Discomfort}_{None}+\beta_{11}{Discomfort}_{Mild}+\beta_{12}{Discomfort}_{Moderate}+\beta_{13}{Discomfort}_{Persistent}+\beta_{14}Lifespan+\beta_{15}Cost$$

*V_j_ =* observable component of utility of dental restoration

*α*ASC = alternative specific constant, a random normally distributed parameter which reflects the observable utility of choosing a treatment option versus choosing no treatment.

*β*_1-8,10-13_ = categorical variables

*β*_9,14-15_ = continuous variables

*β* = coefficient (value) of each attribute level for categorical variables, and the coefficient of changing a continuous variable by one unit in the units of measurement for each categorical variable. For example with lifespan, the beta represents the value of increasing the longevity of a restoration by one year.

**Appendix Figure 3**

**
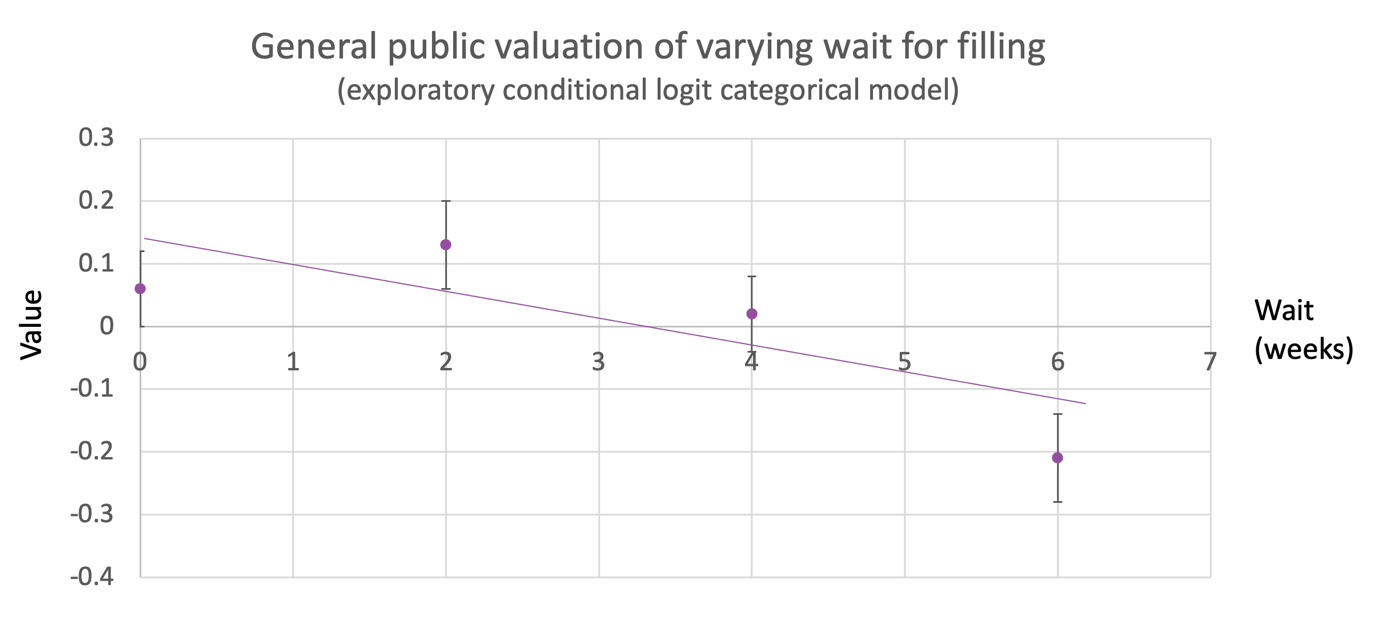
**

**Appendix Figure 4**

**
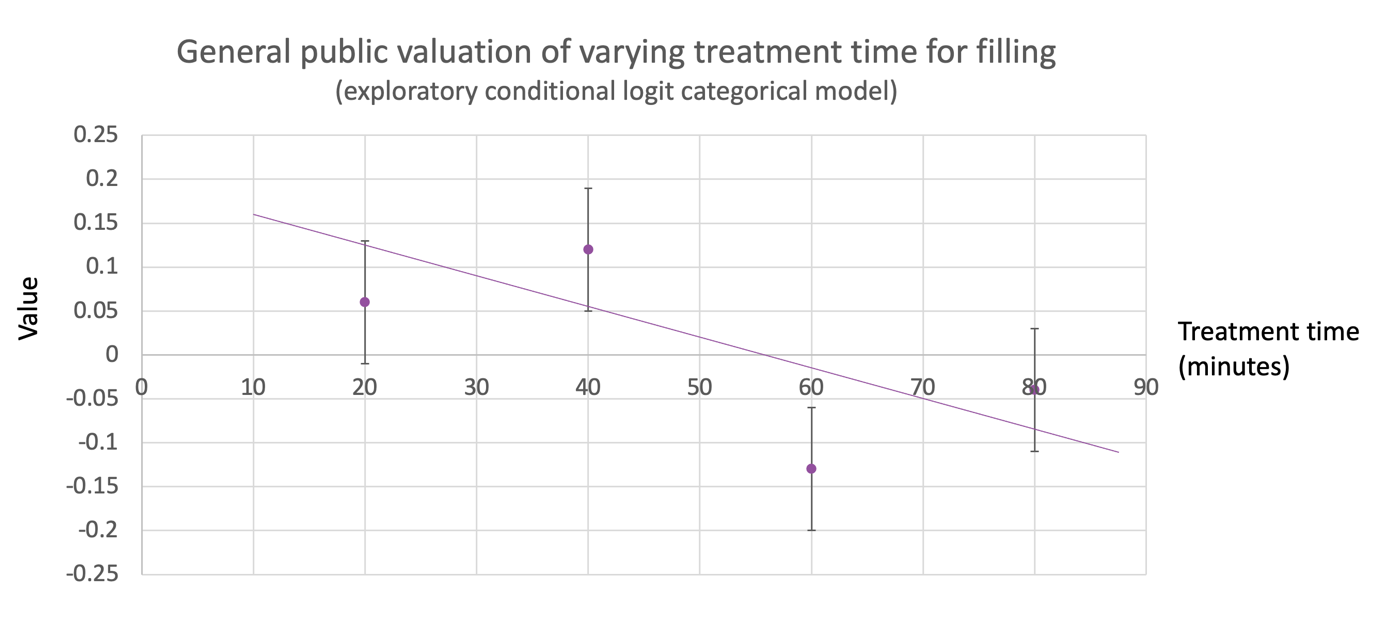
**

**Appendix Figure 5**

**
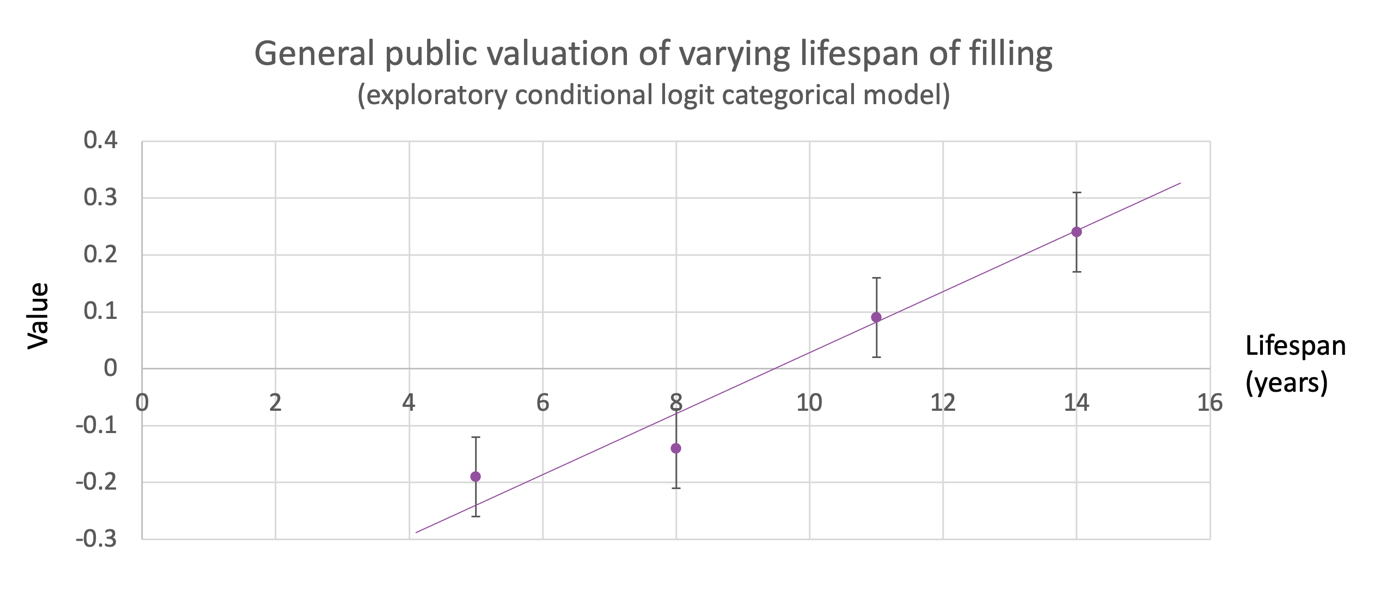
**

**Appendix Figure 6**

**
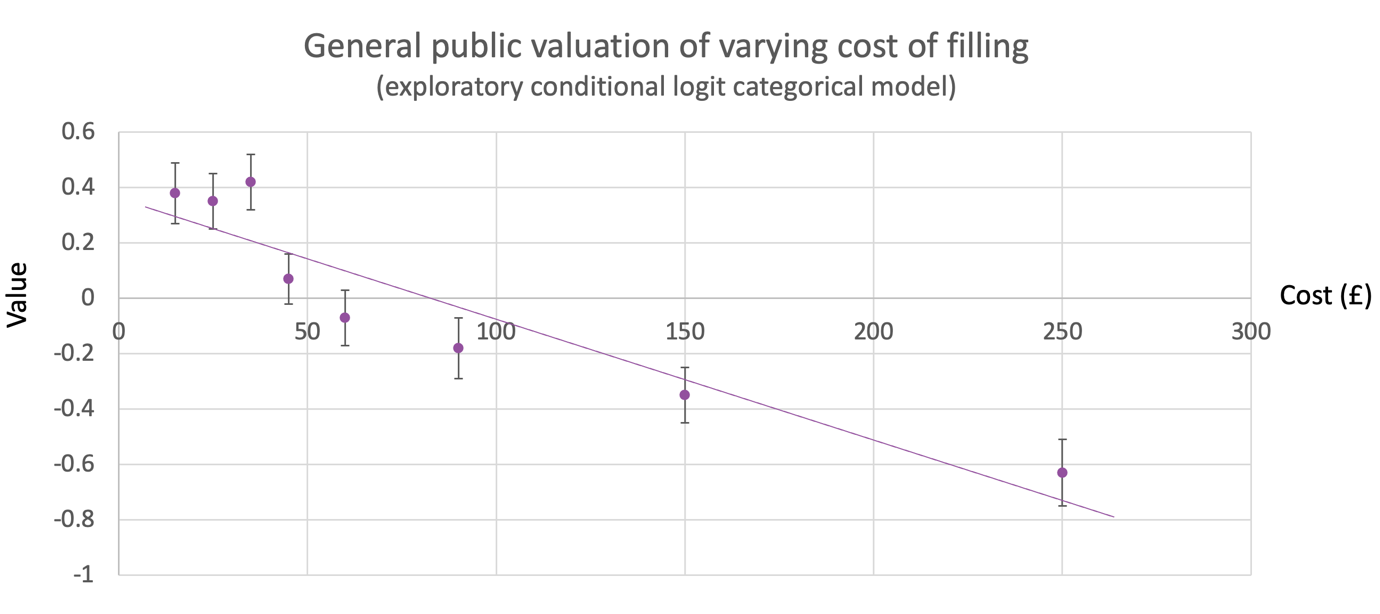
**

**Appendix Figure 7**

Overall UK population marginal willingness to pay for direct posterior restoration attribute levels

**Appendix Table 1. Mixed logit model results showing preferences and willingness to pay for restoration attributes by income sub-groups**

| Attribute | Level | **Low income (n = 221) (observations = 10,608)** | | | | **Higher income (n = 727) (observations = 34,896)** | | | |
| --- | --- | --- | --- | --- | --- | --- | --- | --- | --- |
|  |  | Beta | | mWTP (£) | | Beta | | mWTP (£) | |
|  |  | Mean | SD | Mean | 95% CI | Mean | SD | Mean | 95% CI |
| Waiting time for filling (weeks) | 0^a^ | -0.024 | - | -1.98 | -16.17 - 12.20 | -0.006 | - | -0.76 | -13.31 - 11.78 |
|  | 2 | 0.271** | 0.024 | 22.51 | 6.10 - 38.92 | 0.139* | 0.019 | 18.24 | 4.09 - 32.38 |
|  | 4 | -0.042 | 0.098 | -3.52 | -19.94 - -12.91 | 0.030 | 0.019 | 3.95 | -10.35 - 18.24 |
|  | 6 | -0.204 | 0.024 | -17.01 | -34.97 - -0.95 | -0.164** | 0.032 | -21.42 | -37.14 - -5.71 |
| Clinician | Dentist^a^ | 0.062* | - | 5.15 | 0.89 - 9.42 | 0.056** | - | 7.39 | 4.03 - 10.74 |
|  | Therapist | -0.062* | 0.068** | -5.15 | -9.42 - -0.89 | -0.056** | 0.026 | -7.39 | -10.74 - -4.03 |
| Colour | Silvery grey^a^ | -0.107** | - | -8.90 | -14.53 - -3.27 | -0.192** | - | -25.15 | -30.35 - -19.95 |
|  | White | 0.107** | 0.289** | 8.90 | 3.27 - 14.53 | 0.192** | 0.330** | 25.15 | 19.95 - 30.35 |
| Treatment time^b^ | Per minute | -0.004** | 0.000 | -0.32 | -0.53 - -0.12 | -0.002** | 0.001 | -0.24 | -0.40 - -0.09 |
| Likely discomfort | None^a^ | 0.418** | - | 34.75 | 21.92 - 47.57 | 0.373** | - | 48.73 | 39.10 - 58.37 |
|  | Mild | 0.412** | 0.007 | 34.27 | 23.94 - 44.60 | 0.359** | 0.014 | 46.89 | 38.60 - 55.18 |
|  | Moderate | -0.179** | 0.022 | -14.91 | -24.05 - -5.77 | -0.164** | 0.003 | -21.46 | -28.93 - -13.99 |
|  | Persistent | -0.650** | 0.808** | -54.11 | -69.55 - -38.67 | -0.567** | 0.721** | -74.16 | -86.51 - -61.82 |
| Average lifespan^b^ | Per year | 0.037** | 0.027 | 3.10 | 1.67 - 4.54 | 0.049** | 0.047** | 6.35 | 5.06 - 7.65 |
| Cost^b^ | Per pound | -0.012** | 0.013** | - | - | -0.008** | 0.008** | - | - |
| ASC | Treatment | 3.790** | 2.986** | 315.33 | 225.46 - 405.20 | 3.866** | 2.719** | 505.51 | 419.70 - 591.32 |
|  | No treatment^a^ | -3.790** | - | -315.33 | -405.20 - -225.46 | -3.866** | - | -505.51 | -591.32 - -419.70 |
| Log likelihood | | -2076.65 | | | | -7385.413 | | | |
| AIC | | 4177.30 | | | | 14794.83 | | | |
| BIC | | 4264.53 | | | | 14896.35 | | | |

mWTP, marginal willingness to pay; SD, standard deviation; CI, confidence interval; ASC, alternative specific constant; AIC, Akaike information criterion; BIC, Bayesian information criterion.

^a^Categorical reference level (in effects coded model); ^b^Continuously modelled attribute; *P<0.05; **P≤0.01.

The beta coefficients should not be compared between the two groups because of potential scale heterogeneity. The mWTP values can be compared however as they have been normalised. mWTP = -(beta attribute^b^ or level/beta cost).

**Appendix Table 2. Relative attribute importance: overall and by income**

| Attribute | Overall (n=1002) | | Low income (n=221) | | Higher income (n=727) | |
| --- | --- | --- | --- | --- | --- | --- |
|  | Range beta (+/- 95% CI) | RAI (%) | Range beta (+/- 95% CI) | RAI (%) | Range beta (+/- 95% CI) | RAI (%) |
| Waiting time for filling | 0.340 (0.196) | 7.7 (4.4) | 0.475 (0.404) | 9.0 (7.7) | 0.303 (0.227) | 7.4 (5.5) |
| Clinician | 0.116 (0.043) | 2.6 (1.0) | 0.124 (0.099) | 2.4 (1.9) | 0.113 (0.049) | 2.8 (1.2) |
| Colour | 0.358 (0.060) | 8.1 (1.4) | 0.214 (0.129) | 4.1 (2.5) | 0.385 (0.069) | 9.4 (1.7) |
| Treatment time | 0.142 (0.032) | 3.2 (0.7) | 0.233 (0.142) | 4.4 (2.7) | 0.112 (0.070) | 2.7 (1.7) |
| Likely discomfort | 1.003 (0.134) | 22.8 (3.0) | 1.068 (0.317) | 20.3 (6.0) | 0.940 (0.150) | 23.0 (3.7) |
| Average lifespan | 0.422 (0.065) | 9.6 (1.5) | 0.336 (0.147) | 6.4 (2.8) | 0.437 (0.078) | 10.7 (1.9) |
| Cost | 2.024 (0.183) | 45.9 (4.2) | 2.824 (0.502) | 53.5 (9.5) | 1.797 (0.202) | 44.0 (4.9) |

RAI, relative attribute importance; CI, confidence interval.
